# Monitoring treatment effects in lung cancer-bearing mice: clinical CT and clinical MRI compared to micro-CT

**DOI:** 10.1186/s41747-020-00160-7

**Published:** 2020-05-13

**Authors:** Judith E. Spiro, Miriam Rinneburger, Dennis M. Hedderich, Mladen Jokic, Hans Christian Reinhardt, David Maintz, Moritz Palmowski, Thorsten Persigehl

**Affiliations:** 1 Department of Diagnostic and Interventional Radiology, University Hospital of Cologne, Kerpener Str. 62, 50937 Cologne, Germany; 2 Department of Radiology, University Hospital, LMU Munich, Marchioninistr. 15, 81377 Munich, Germany; 3 Department of Neuroradiology, Klinikum rechts der Isar, Technical University of Munich, School of Medicine, Ismaninger Str. 22, 81675 Munich, Germany; 4 Cologne Excellence Cluster on Cellular Stress Response in Aging-Associated Diseases, Joseph-Stelzmann-Str. 26, 50931 Cologne, Germany; 5 Department of Internal Medicine, Division I, Hematology/Oncology, University Hospital of Cologne, Kerpener Str. 62, 50937 Cologne, Germany; 6 Center for Molecular Medicine Cologne, University of Cologne, Robert-Koch-Straße 21, 50931 Cologne, Germany; 7 Institute of Experimental Molecular Imaging, University Aachen, Forckenbeckstr. 55, 52074 Aachen, Germany; 8 Radiology Baden-Baden, Beethovenstr. 2, 76530 Baden-Baden, Germany

**Keywords:** Cisplatin, Lung neoplasms, Magnetic Resonance Imaging, Mice, X-ray microtomography

## Abstract

**Background:**

Compared to histology-based methods, imaging can reduce animal usage in preclinical studies. However, availability of dedicated scanners is limited. We evaluated clinical computed tomography (CT) and magnetic resonance imaging (MRI) in comparison to dedicated CT (micro-CT) for assessing therapy effects in lung cancer-bearing mice.

**Methods:**

Animals received cisplatin (*n* = 10), sham (*n* = 12), or no treatment (*n* = 9). All were examined via micro-CT, CT, and MRI before and after treatment. Semiautomated tumour burden (TB) calculation was performed. The Bland-Altman, receiver operating characteristic (ROC), and Spearman statistics were used.

**Results:**

All modalities always allowed localising and measuring TB. At all modalities, mice treated with cisplatin showed a TB reduction (*p* ≤ 0.012) while sham-treated and untreated individuals presented tumour growth (*p* < 0.001). Mean relative difference (limits of agreement) between TB on micro-CT and clinical scanners was 24.7% (21.7–27.7%) for CT and 2.9% (−4.0–9.8%) for MRI. Relative TB changes before/after treatment were not different between micro-CT and CT (*p* = 0.074) or MRI (*p* = 0.241). Mice with cisplatin treatment were discriminated from those with sham or no treatment at all modalities (*p* ≤ 0.001). Using micro-CT as reference standard, ROC areas under the curves were 0.988–1.000 for CT and 0.946–0.957 for MRI. TB changes were highly correlated across modalities (*r* ≥ 0.900, *p* < 0.001).

**Conclusions:**

Clinical CT and MRI are suitable for treatment response evaluation in lung cancer-bearing mice. When dedicated scanners are unavailable, they should be preferred to improve animal welfare.

## Key points

- Easy-to-apply protocols allowed reliable discrimination between treated and untreated lung cancer-bearing mice on both micro-computed tomography (micro-CT) and clinical CT or clinical magnetic resonance imaging (MRI) scanners.
- Volumetry of tumour burden on clinical scanners correlated well with micro-CT measurements.
- Clinical CT and MRI scanners are suitable for response evaluation in lung cancer-bearing mice.

## Background

The rising incidence of lung cancer over the past century as well as its high morbidity and mortality rates has made this disease an important topic for clinical and preclinical research [1]. Focus has been recently placed on the evaluation of targeted therapies, which take into account the high genetic variability of lung tumours caused by driver gene mutations and markedly improve therapy outcome [2]. The preclinical phase of drug development depends on medication testing in animal models, mostly mice. In this context, the harms to the animals have to be carefully weighed against the potential benefits of an experiment.

Unlike histology-based methods, which require surgery or mice sacrifice, *in vivo* imaging allows for non-invasive evaluation of therapy response and thereby permits a reduction of animal numbers compared to studies relying on histology as sole method for response evaluation [3, 4]. Most preclinical studies use micro-computed tomography (micro-CT) or dedicated small animal magnetic resonance (micro-MRI) scanners to visualise and quantify lung tumours in mice, because both modalities offer high resolution and good correlation with histopathological results [5, 6]. However, micro-CT examinations go along with significant radiation exposure, which could cause unintentional treatment effects [7]. Additionally, small animal scanners, especially micro-MRI, are not available in all research centres, primarily due to high acquisition and maintenance costs. Clinical CT and magnetic resonance imaging (MRI) scanners, by contrast, are widely available, especially in hospitals large enough to conduct preclinical research. Previous studies have shown that imaging of murine lungs using clinical CT and MRI scanners is possible [8–10].

The aim of our study was to assess the suitability of clinical CT and MRI for the evaluation of tumour response in a lung cancer mouse model and to propose easy-to-apply protocols for both modalities to facilitate tumour monitoring in preclinical trials.

## Methods

We performed a prospective preclinical study, in which treatment response of lung cancer-bearing mice assessed by clinical CT and clinical MRI was compared to the results of micro-CT measurements.

### Mouse model

Thirty-one mice with lung tumours were included in the study. Tumour development was driven by the expression of oncogenic *Kras* and the deletion of *Tp53* and *Ercc1*, as described in detail by Jokic et al. [11]. Tumour-bearing mice were divided into three arms: mice receiving three cycles of cisplatin therapy (intraperitoneal injections of 7.5 mg/kg of body weight equating 7.5 mL/kg of body weight of cisplatin diluted in phosphate-buffered saline (PBS), one injection per week in three consecutive weeks) (*n* = 10), mice receiving three cycles of sham treatment (intraperitoneal injections of 7.5 mL/kg of body weight of PBS, one injection per week in three consecutive weeks) (*n* = 12), and mice which did not receive any therapy at all (*n* = 9). All mice were examined via micro-CT, clinical CT, and clinical MRI 2 days before and 24 days after treatment initiation. All animal experiments were performed in accordance with the national and European regulations and were approved by the local authorities (Landesamt für Natur, Umwelt und Verbraucherschutz NRW, reference number NRW 84-02.04.2013.A136).

### Imaging

All mice underwent inhalation anaesthesia with isoflurane (2.0 to 2.5%) in air during image acquisition. Clinical MRI, clinical CT, and micro-CT scans were performed 2 days before and 24 days after treatment initiation, respectively. In order to keep the imaging protocols simple and to minimise stress and health risks for the mice, we decided to forego application of intravenous contrast media and respiratory gating.

#### Micro-CT

The LaTheta LCT-100A micro-CT-scanner (Aloka Co., Tokyo, Japan) was used with the following parameters: 50 kVp x-ray source with a focal spot size of 50 μm, 1 mA current, 4,340 ms exposure time per projection, 480 × 480 matrix covering a 49.48 × 49.48 mm^2^ field of view, and 0.3 mm voxel spacing in the *z*-axis, resulting in a voxel size of 0.1 × 0.1 × 0.3 mm^3^. Phantom measurements conducted by Stiller et al. [12] in the LaTheta LCT-100A revealed a radiation exposure of about 5 mGy in scans consisting of 58 slices. Micro-CT examinations performed in our study consisted of 40 to 60 slices, depending on the murine lung size.

#### Clinical CT

Imaging was performed on a Brilliance iCT 256-slice scanner (Philips Healthcare, Best, The Netherlands) in prone position using the following parameters: tube voltage 120 kV, tube current 100 mAs, slice thickness 0.67 mm, matrix 1,024 × 1,024, field of view as small as possible to include the murine thorax, rotation time 0.4 s, and pitch 0.4. Volume CT dose index for one slice was 13.6 mGy (calculated using a 16-cm human head phantom). The scan length fluctuated between approximately 1.5 and 2 cm, depending on the murine lung size, resulting in a dose length product of approximately 20.4 to 27.2 mGy × cm. Images were reconstructed using a hard reconstruction kernel.

#### Clinical MRI

Scans were acquired on an Ingenia 3.0-T system (Philips Healthcare, Best, The Netherlands) combined with a commercially available small animal coil (Philips Research, Hamburg, Germany) with heating function to preserve body temperature during the examination. The protocol consisted of one axial multi-shot T2-weighted turbo-spin-echo (TSE) sequence with the following parameters: echo time 65 ms, repetition time 1,540 ms, flip angle 90°, field of view 40 × 40 mm, matrix 256 × 256, slice thickness 1 mm without gap, acquired voxel size 0.19 × 0.27 × 1 mm, and reconstructed voxel size 0.15 × 0.15 × 1 mm, number of signal acquisitions 6. Standard scan time for 15 slices was 5:05 min. The detailed scan protocol of the T2-weighted sequence is available at the supplement for all vendors, as well as an ExamCard for Philips 3.0T Achieva and Ingenia MRI systems ready to use.

### Image analysis

**Fig. 1:**
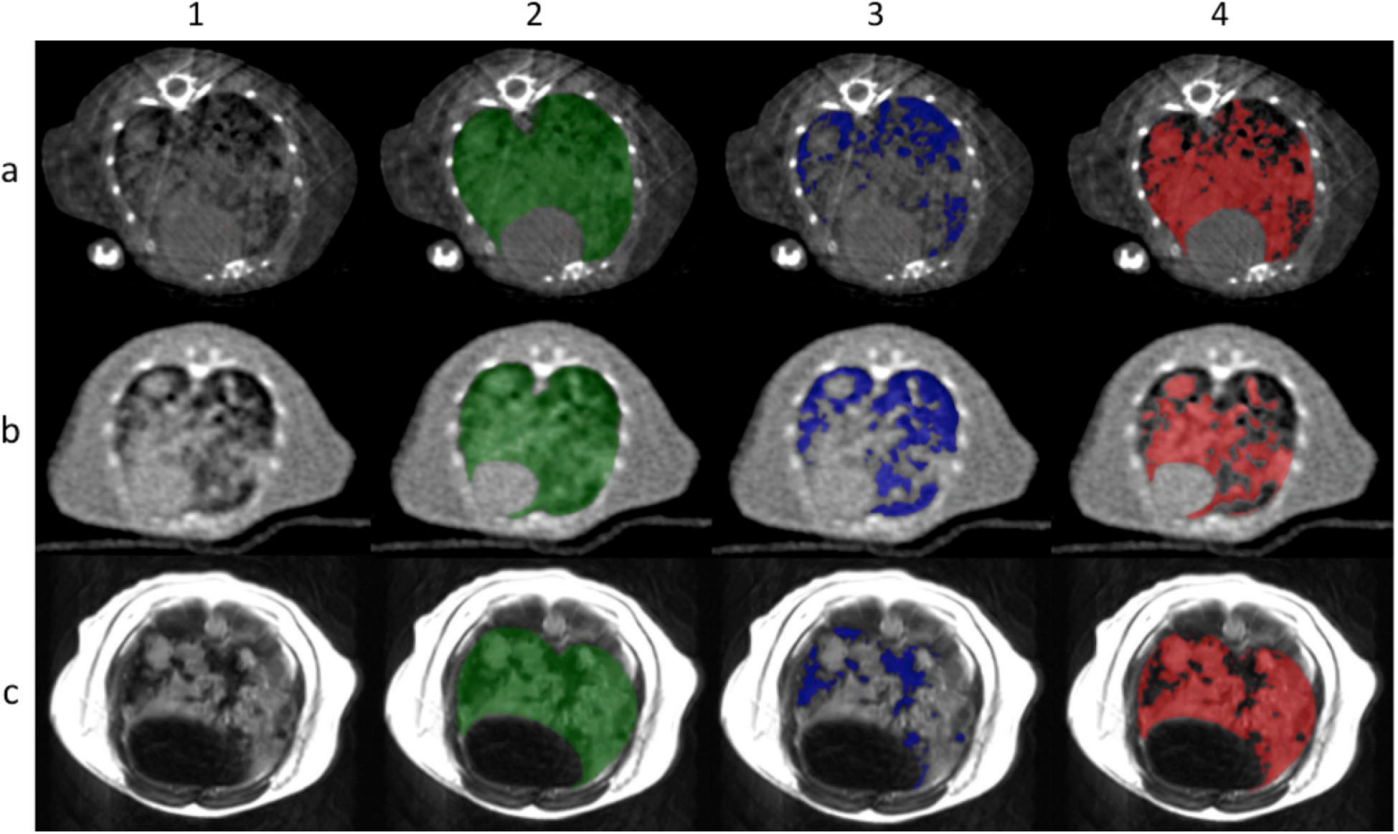
Overview of the segmentation process. Segmentation of micro-CT (row **a**), clinical CT (row **b**), and clinical MRI (row **c**) scans. After manual definition of the lungs (column 2), ventilated tissue (column 3) and tumour tissue (column 4) were semiautomatically segmented via a threshold-based algorithm. CT, Computed tomography; MRI, Magnetic resonance imaging

Total tumour burden was quantified by semiautomated, threshold-based volumetry using the postprocessing software IntelliSpace Discovery Imalytics Fundamentals (Philips Healthcare, Best, The Netherlands). At first, mouse lungs were manually defined excluding the mediastinal structures. In a second step, semiautomated tumour segmentation was performed using a threshold-based algorithm. For definition of the thresholds, two radiologists with 13-year (T.P.) and 2-year (J.E.S.) experience in rodent imaging visually evaluated the segmentation results of different threshold settings. They found the optimal threshold values between ventilated lung and soft tissue: −200 HU on micro-CT and clinical CT scans and 200 arbitrary units on MRI. Ventilated lung tissue showed lower and tumours showed higher HU and arbitrary units than the threshold, respectively. Figure 1 gives an overview of the segmentation process.

On micro-CT and clinical CT images, tumours show higher density values than ventilated lung tissue but similar density to intrapulmonary vessels. Hence, intrapulmonary vessels were included in the tumour volume on micro-CT and clinical CT datasets. In contrast, on clinical T2-weighted MRI scans, ventilated lung tissue and vessels appear hypointense due to low proton density and flow void effects, respectively, whereas tumours show high signal intensity. Non-malignant pulmonary processes, such as oedema, atelectasis, and pneumonia, were included in the tumour volume on micro-CT and clinical CT as well as on MRI scans.

### Statistical analysis

Statistical analysis was performed using SPSS (IBM Corporation, Armonk, NY, USA) version 25. Normal distribution of data was assessed by the Shapiro-Wilk test. To assess differences of tumour volumes before and after treatment as measured by the three modalities, paired *t* tests or Wilcoxon’s signed-rank tests were performed depending on whether data was normally distributed or not. We used micro-CT as a reference standard for assessing tumour size and created the Bland-Altman plots comparing tumour volumes measured by CT and MRI, respectively, to compare the different measurements [13]. In order to evaluate the mean relative difference of tumour volumes measured by both clinical scanners and micro-CT, respectively, pre- and posttreatment data were combined. To evaluate the change of lung tumour burden, the ratio of posttreatment tumour volume/pretreatment tumour volume was calculated for clinical CT, MRI, and micro-CT.

Receiver operating characteristic (ROC) analyses were performed in a step-wise manner. First, we examined how well changes of tumour volume measured by all three modalities were able to discriminate the treatment group (cisplatin) from the non-treatment groups (PBS and no treatment, respectively). Mice with sham and without treatment were combined in order to increase the number of individuals in this group and thereby make the analysis more robust and clear. Second, we measured how well clinical CT and MRI could differentiate between treatment responders and non-responders as determined by micro-CT volume reductions of 10%, 15%, 20%, and 30%. Relative tumour sizes (*i.e*, posttreatment volume/pretreatment volume) determined by CT and MRI, respectively, were used as test variables. To investigate the correlation of tumour volume changes between all three modalities, Spearman’s *r* was calculated. All tests were two-sided. The alpha level was at 0.05.

## Results

### Tumour detection

Micro-CT, clinical CT, and clinical MRI enabled to localise and measure tumour burden before and after treatment in all examinations, resulting in *n* = 186 imaging studies in total.

### Volume measurements across modalities and treatment groups

Measurements of absolute tumour and lung volumes were normally distributed as determined by the Shapiro-Wilk test, except for pretreatment tumour volume in the PBS group. All mice treated with cisplatin (*n* = 10) showed a significant reduction of tumour burden in all modalities (reduction at micro-CT 0.237 ± 0.236 (mean ± standard deviation), *p* = 0.011; reduction at CT 0.212 ± 0.213, *p* = 0.012; reduction at MRI 0.323 ± 0.326; *p* = 0.012). Sham-treated individuals in the PBS group (*n* = 12) and those without treatment (*n* = 9) presented significant tumour growth (PBS group: growth at micro-CT 0.677 ± 0.239, *p* < 0.001; growth at CT 0.580 ± 0.180, *p* < 0.001; growth at MRI 0.875 ± 0.216, *p* < 0.001; no treatment group: growth at micro-CT 0.516 ± 0.156, *p* < 0.001; growth at CT 0.525 ± 0.161, *p* < 0.001; growth at MRI 0.893 ± 0.262, *p* < 0.001). Detailed information about absolute tumour volumes as well as absolute lung volumes in different modalities across all three groups is reported in Table 1.

**Table 1:** Absolute measurements of tumour volume and lung volume as measured by micro-CT, clinical CT, and MRI for all three treatment groups

			Micro-CT		Clinical CT		Clinical MRI	
			T0	T1	T0	T1	T0	T1
Cisplatin (*n* = 10)	Lung volume (mL)	Mean	0.959	0.794	0.964	0.788	0.987	0.724
		SD	0.235	0.204	0.224	0.171	0.336	0.234
	Tumour volume (mL)	Mean	0.656	0.418	0.505	0.293	0.724	0.401
		SD	0.291	0.180	0.244	0.148	0.398	0.225
PBS (*n* = 12)	Lung volume (mL)	Mean	0.724	1.284	0.688	1.220	0.599	1.346
		SD	0.157	0.305	0.172	0.243	0.166	0.287
	Tumour volume (mL)	Mean/median	0.301*	1.059	0.262	0.842	0.278	1.153
		SD/IQR	0.240–0.610**	0.334	0.128	0.282	0.167	0.343
No treatment (*n* = 9)	Lung volume (mL)	Mean	1.087	1.597	1.080	1.497	0.988	1.736
		SD	0.238	0.289	0.198	0.269	0.230	0.235
	Tumour volume (mL)	Mean	0.782	1.298	0.594	1.119	0.683	1.576
		SD	0.243	0.206	0.191	0.217	0.260	0.195

*CT* Computed tomography, *IQR* Interquartile range, *MRI* Magnetic resonance imaging, *PBS* Phosphate-buffered saline, *SD* Standard deviation, *T0* Pretreatment, *T1* Posttreatment. In case of non-normally distributed data, median (*) and IQR (**) are given instead and marked

**Fig. 2:**
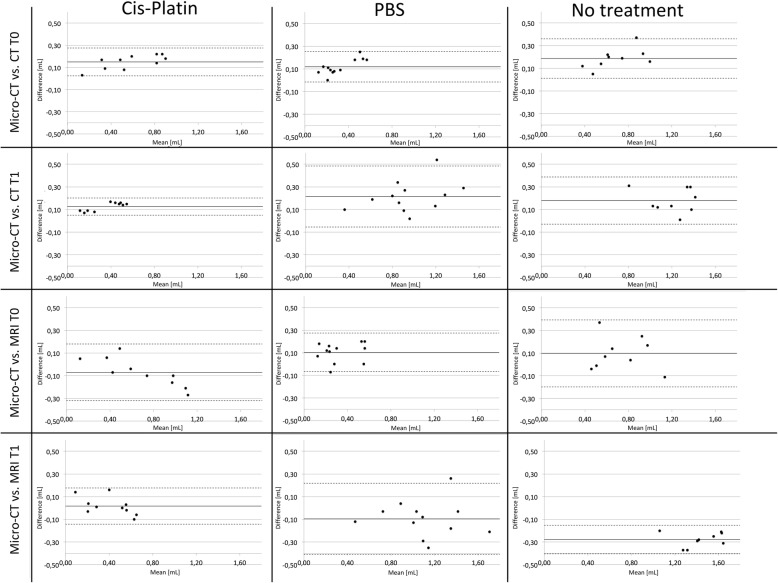
Bland-Altman plots to compare tumour volumes measured by micro-CT, CT, and MRI. Bland-Altman plots were created comparing CT-derived (first and second row) and MRI-derived (third and fourth row) measurements of tumour volume to micro-CT, which served as gold standard. *y*-axis shows the difference (mL), and *x*-axis shows the mean [mL] of the two measurements compared. Horizontal lines are added at the mean (continuous line) of the difference and at ± 1.96 standard deviations (dotted lines). CT, Computed tomography; MRI, Magnetic resonance imaging; T0, Pretreatment; T1, Posttreatment

The Bland-Altman comparison between pretreatment and posttreatment MRI- and CT-derived tumour volumes and the reference standard as derived from micro-CT is shown in Fig. 2. Overall, good correlation between measurements could be observed. Three outliers above 1.96 standard deviations of the mean difference were observed in the PBS group and one in the untreated group. Differences of all measurements in the cisplatin group were within ± 1.96 standard deviations of the mean difference. Mean relative difference between tumour volumes measured on micro-CT and CT images was 24.7%, and the limits of agreement ranged from 21.7 to 27.7%. Between micro-CT and MRI volumetric measurements, mean relative difference was 2.9% with limits of agreement from −4.0 to 9.8%.

### Treatment monitoring

**Fig. 3:**
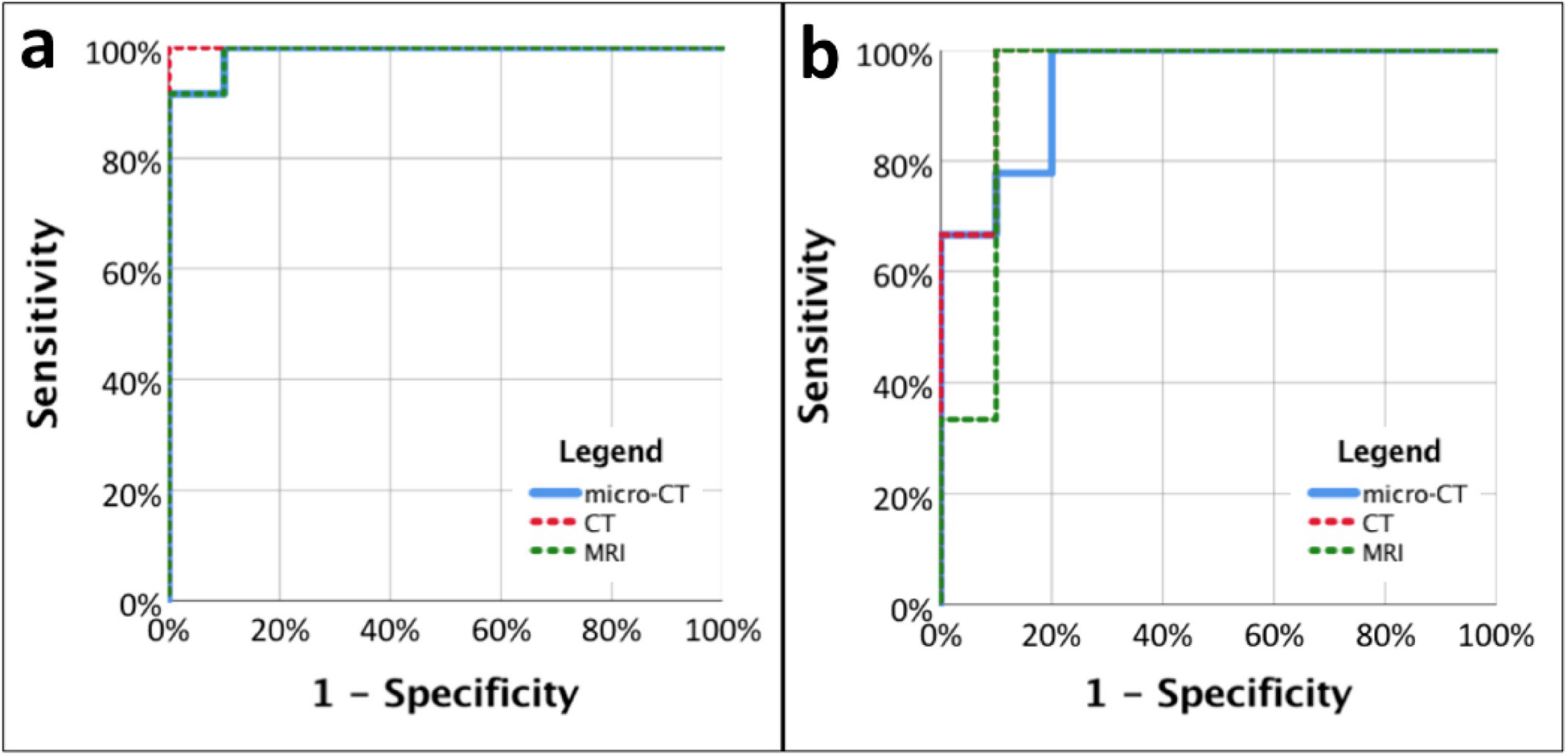
Receiver operating characteristic analysis for assessing the ability of tumour volume changes measured by micro-CT, CT, and MRI to discriminate between mice that received treatment and mice that received sham (**a**) or no treatment (**b**). Areas under the curve (AUC) were significant for all three modalities. **a** Cisplatin *versus* phosphate-buffered saline: micro-CT (blue line), AUC 0.902, *p* < 0.001; CT (red dashed line), AUC 1.0, *p* < 0.001; MRI (green dashed line), AUC 0.992, *p* < 0 .001. **b** Cisplatin *versus* no treatment: micro-CT (blue line), AUC 0.944, *p* = 0.001; CT (red dashed line), AUC 0.967, *p* = 0.001; MRI (green dashed line), AUC 0.933, *p* = 0.001). CT, Computed tomography; MRI, Magnetic resonance imaging

Measurements of relative changes in tumour volume measured by MRI and micro-CT were not normally distributed as determined by the Shapiro-Wilk test. Relative changes in tumour volume between pretreatment and posttreatment measurements were not significantly different between micro-CT and CT (*p* = 0.074) and micro-CT and MRI (*p* = 0.241), respectively. At ROC analysis (Fig. 3), mice with cisplatin treatment were discriminated well from mice with sham treatment and from mice without treatment by relative tumour volume reductions derived by all three modalities. For cisplatin *versus* PBS, the area under the curve (AUC) of micro-CT was 0.902 (*p* < 0.001), that of CT 1.0 (*p* < 0.001), and that of MRI 0.992 (*p* < 0.001). For cisplatin *versus* no treatment, the AUC of micro-CT was 0.944 (*p* = 0.001), that of CT 0.967 (*p* = 0.001), and that of MRI 0.933 (*p* = 0.001).

At additional ROC analyses using micro-CT as a reference standard, the two clinical modalities discriminated well between mice, which received chemotherapy, and animals with sham or no treatment. Derived AUC values were highly significant for both CT and MRI at all thresholds. Adopting the 10% tumour volume reduction threshold, the CT AUC was 0.989 (95% confidence interval (CI) 0.961–1.000, *p* < 0.001) and the MRI AUC was 0.957 (CI 0.873–1.000, *p* < 0.001). Adopting the 15% tumour volume reduction threshold, the CT AUC was 0.989 (CI 0.961–1.000, *p* < 0.001) and the MRI AUC was 0.957 (CI 0.873–1.000, *p* < 0.001). Adopting the 20% tumour volume reduction threshold, the CT AUC was 0.988 (CI 0.957–1.000, *p* < 0.001) and the MRI AUC was 0.946 (CI 0.862–1.000, *p* < 0.001). Adopting the 30% tumour volume reduction threshold, the CT AUC was 1.000 (CI 1.000–1.000, *p* < 0.001) and the MRI AUC was 0.953 (CI 0.874–1.000, *p* < 0.001).

Tumour volume changes were highly correlated across all three modalities as determined by Spearman’s correlation. For CT *versus* micro-CT, *r* was 0.957 (*p* < 0.001); for MRI *versus* micro-CT, *r* was 0.900 (*p* < 0.001); and for CT *versus* MRI, *r* was 0.921 (*p* < 0.001).

## Discussion

Our study reveals that clinical CT and MRI are suitable for treatment response evaluation in mice with lung cancer. Both modalities as well as the reference standard micro-CT imaging showed significant reduction of tumour burden in treated (cisplatin) and significant cancer growth in untreated (sham and no therapy) mice. Comparison of absolute tumour volumes determined by clinical CT and MRI to the results of micro-CT measurements demonstrated good correlation. Also, relative changes in tumour volume before and after treatment were not significantly different between the reference standard micro-CT and the clinical scanners and highly correlated across all thee modalities. Finally, assessment of relative tumour volume reduction allowed reliable discrimination between treated and untreated mice on micro-CT as well as on clinical CT and MRI scans. Limits of agreements for tumour volume ranged from 21.7 to 27.7% between micro-CT and CT and from −4.0 to 9.8% between micro-CT and MRI.

One possible explanation for the mean relative difference of 24.7% between tumour volumes measured on micro-CT and CT scans is the beam hardening artefacts on micro-CT images, which are known to influence quantitative imaging [14]. In the literature on variation of semiautomated lung nodule volumetry between CT scans, limits of agreement of up to −35.4 to 28.6% are reported [15]. We therefore consider our results acceptable in the context of preclinical studies on lung cancer treatment.

The scan protocols we used to visualise murine tumour burden are simple and can easily be transferred to any clinical CT or MRI scanner. To facilitate imaging, we forewent respiratory gating, a tool that can prevent breathing-induced blurring and is hence very useful for image quality optimisation [16, 17]. However, several studies proved that non-respiratory gated imaging of rodent lungs delivers reliable results [8–10, 18]. The very small range of respiratory motion in mice, which is on the same scale as the resolution limit of the scanners, explains this phenomenon [10]. To keep the imaging protocols simple, we also forewent the use of intravenous contrast agent, which is essential for lung cancer staging in humans [19]. In mice, however, commonly used iodinated contrast media have a very short biological half-life and therefore do not improve image quality [20]. Although new techniques have been described to overcome this limitation, the extent to which they improve the accuracy of volumetric tumour measurement is still unknown [16, 21]. Moreover, venous puncture and injection of contrast media expose mice to high levels of stress and possible health risks [20]. Beyond that, as every substance could theoretically impact physiological processes, administration of contrast media should, if possible, be avoided in drug development studies to prevent corruption of results [22].

We performed semiautomated, threshold-based volumetry to quantify tumour burden. One limitation of this method is the lacking discrimination between different kinds of soft tissue due to their similar densities or signal intensities. Pulmonary vessels, non-malignant pulmonary processes, and necrosis are hence included in the tumour volume [23, 24]. Besides application of contrast agent, one approach to avoid this bias is manual segmentation of tumour burden, which has been shown to be well applicable in mice with large, discrete neoplasms [25]. Our mouse model, however, develops multifocal, irregularly shaped tumour nodules, which are not reliably measurable by manual segmentation [23]. For these tumour types, quantitative volumetry of tumour burden from non-gated, unenhanced micro-CT imaging has been shown to strongly correlate with histological analysis in lung cancer-bearing mice [18, 23]*.* This is explained by the fact that pulmonary vessels and benign pulmonary processes only make a relatively small portion of the soft tissue. Their inclusion in the tumour volume does hence not strongly affect therapy response evaluation [23].

Rodent experiments are an essential component of preclinical studies. In the past decades, special focus has been placed on minimising animal use and suffering. In particular, longitudinal *in vivo* imaging has been proven to be a reliable method for response evaluation in mice with lung cancer [4, 5, 18, 23, 26, 27]. Dedicated small animal CT and MRI scanners were designed for this purpose, but their availability is limited due to relatively high acquisition and maintenance costs. As clinical scanners are widely available, their utilisation in place of histology-based methods could strongly contribute to animal welfare and reduce the number of animals used in preclinical studies [4].

The relatively small number of mice is a limitation of our study, which reduces the statistical power of the analyses. As elaborated above, when using live animals for academic research, the harms put upon them and the potential benefits of an experiment have to be carefully weighed. Considering this and the unambiguous results of our study, we think that the number of mice we used is reasonable and justifiable.

When considering which modality to choose for response evaluation in preclinical trials, the fact that clinical CT and micro-CT expose scanned individuals to ionising radiation has to be taken into account. This could induce unintentional treatment effects and hereby hamper the results of response evaluation in mice [7]. Therefore, non-ionising MRI might be particularly well suited in longitudinal studies, where treatment response is evaluated at different time points and treatment effects due to radiation should be avoided.

In summary, our study reveals that clinical MRI and CT scanners are well suited for follow-up and response evaluation in mice with lung cancer in the course of preclinical studies. Further studies, preferably including micro-MRI, are desirable to substantiate our findings.

## Data Availability

The datasets analysed during the current study are available from the corresponding author on reasonable request.

## Abbreviations

AUC: Area under the curve
CT: Computed tomography
Micro-CT: Micro-computed tomography
MRI: Magnetic resonance imaging
PBS: Phosphate-buffered saline
ROC: Receiver operating characteristic
